# Human Head and Helmet Interface Friction Coefficients with Biological Sex and Hair Property Comparisons

**DOI:** 10.1007/s10439-023-03332-9

**Published:** 2023-08-04

**Authors:** Nicole E.-P. Stark, Charlotte Clark, Steve Rowson

**Affiliations:** 1 Department of Biomedical Engineering and Mechanics, Virginia Tech, 120 Kelly Hall, 325 Stanger Street, MC 0298, Blacksburg, VA 24061 USA; 2 Department of Materials Science and Engineering, Virginia Tech, Blacksburg, USA

**Keywords:** Friction, Static, Dynamic, Helmet, Headforms

## Abstract

Dummy headforms used for impact testing have changed little over the years, and frictional characteristics are thought not to represent the human head accurately. The frictional interface between the helmet and head is an essential factor affecting impact response. However, few studies have evaluated the coefficient of friction (COF) between the human head and helmet surface. This study’s objectives were to quantify the human head’s static and dynamic COF and evaluate the effect of biological sex and hair properties. Seventy-four participants slid their heads along a piece of helmet foam backed by a fixed load cell at varying normal force levels. As normal force increased, static and dynamic human head COF decreased following power–law curves. At 80 N, the static COF is 0.32 (95% CI 0.30–0.34), and the dynamic friction coefficient is 0.27 (95% CI 0.26–0.28). Biological sex and hair properties were determined not to affect human head COF. The COFs between the head and helmet surface should be used to develop more biofidelic head impact testing methods, define boundary conditions for computer simulations, and aid decision-making for helmet designs.

## Introduction

Linear and rotational head accelerations influence brain injury risk [9, 22, 23, 28, 29], and an emphasis on reducing rotational acceleration has driven new helmet design features. Bike helmet manufacturers have sought to decrease concussion risk by reducing the head’s rotational acceleration upon impact using different rotation-mitigating technologies. These technologies include Multi-directional Impact Protection System (MIPS), *WaveCel*, POC Shearing Pads INside (SPIN), and 6D Omni-Directional Suspension (ODS) [10]. MIPS is a technology that provides a slip plane for the head to slide independently from the helmet shell [7]. POC SPIN technology uses silicone gel-filled pads to decouple the head from the helmet [8]. In another approach, the *WaveCel* technology reduces head rotational acceleration through liner cells flexing and gliding [10]. Like *WaveCel*, the 6D ODS does not use a slip plane but an elastomeric damper array between an inner and outer helmet layer [8]. These new design developments highlight that interfacial properties, such as friction between the helmet and head, play a significant role in impact response. Reducing friction between the headform and helmet correlates with lower head rotational kinematics [1, 13]. Therefore, friction could influence helmet testing and data interpretation when determining injury risk [14].

Despite the technological advances, dummy headforms implemented in head impact testing have changed little over the years and have known limitations to their biofidelity [36]. Of note, the dummy headforms’ friction characteristics are thought to not accurately represent the human head [1, 11, 19, 35]. The Hybrid III headform, developed for automotive crash testing but now also used in helmet testing, has a vinyl plastisol skin with a high friction coefficient [19, 35]. Some test methods cover the Hybrid III headform with a stocking to reduce friction and better simulate the human head’s friction characteristics [12, 32, 34]. Other helmet test methods use a National Operating Committee on Standards for Athletic Equipment (NOCSAE) headform. The NOCSAE headform was developed exclusively for helmet testing, and its outer layer is composed of polyurethane skin [11, 16, 18]. Magnesium headforms, which are also commonly used in standards for helmet testing, have no outer layer and have been reported to have lower coefficients of friction than the Hybrid III [13, 25, 33]. However, few studies have evaluated the coefficient of friction (COF) between the human head and helmet interior surface; therefore, the frictional biofidelity of these headforms is unknown [13, 33].

The most relevant friction study, by Trotta et al., measured the friction between six cadaver heads and a liner material using a 20-mm probe on an Instron [33]. This study found that the human head had a static COF between 0.21 and 0.35 and a dynamic COF between 0.20 and 0.32 for a normal force of 20–200 N, and that hair had no effect [33]. However, the results were limited by the small sample size, only testing cadavers, and a small interacting surface area. Another study by Ebrahimi et al. found the human skin COF against helmet padding to be 0.683 using force measurements from ten trials at two different normal forces [13]. Few details were provided describing the methods used to calculate COF. Ebrahimi et al. were limited by not specifying the area of skin, a small sample size, and not evaluating friction as a function of applied normal force instead of averaged across normal forces [13].

Human skin has viscoelastic properties [5, 6, 30] which may deviate from the classic Amonton and Coulomb friction law that indicates friction is proportional to normal force [26]. To assess friction coefficients for human skin accurately, it is essential to consider the normal force since previous studies have demonstrated a decline in dynamic and static friction coefficients as the normal force increases for human skin [30]. However, it should be noted that these studies were conducted on other areas of the skin [30]. Similarly, Trotta et al. observed a reduction in human head COF when higher stroke frequencies (material moving over the head) and increased normal force were applied, indicating that there is a relationship between normal force and COF for the human head [33]. The textile industry observed a comparable effect when evaluating viscoelastic materials and often quantifies this using a power–law relationship [15, 27]. Therefore, a power–law relationship could be necessary in describing the decrease in friction coefficients with increasing normal force when evaluating human head friction.

Our study objective was to evaluate the human head’s static and dynamic friction coefficients against expanded polystyrene (EPS) helmet foam over varying normal force levels. Our approach included a larger sample size, a larger interacting surface area, and living participants, compared to the works of Trotta et al. and Ebrahimi et al. We also evaluated biological sex and hair properties’ effects on friction coefficients. Defining the human head static and dynamic friction coefficients on a helmet surface can be used to develop more biofidelic dummy headforms, describe boundary conditions for computer-aided simulations, and aid decision-making for helmet designs.

## Materials and Methods

We quantified human head friction coefficients using a rigidly mounted 3-axis load cell (Humanetics 2866 seat mount load cell; Farmington Hills, Minnesota, USA) with an EPS foam interface extracted from the crown of a Bell Vert 2.0 Bike/Skate helmet (Vista Outdoor; Rantoul, Illinois, USA). The EPS foam was 10.5 cm by 10.5 cm and had a measured density of 77 kg/m^3^. The EPS foam was securely attached to a plate using adhesive, which was then secured to the load cell interface according to manufacturer specifications. Through an Institutional Review Board-approved protocol, 74 participants were recruited, consented, and attended a single data collection session.

Participants’ height, weight, biological sex, and hair properties (self-reported curl type, style at the time of participation, and tightness of style at participation) were recorded. Curl type was self-reported based on 4 defined categories: tight curls, curly, wavy, and straight. At the time of participation, participants were instructed to wear their hair as if they were going to wear a helmet. The styles at the time of participation included the following: shorter than one inch, longer than one inch but shorter than shoulder length, down, braided (any type of braid was included), bun, or ponytail. All participants that wore a ponytail or bun style tied their hair below the occipital bone indicating a low bun or low ponytail style. The tightness of the style was also recorded at the time of participation. A tight style indicated that the hair was tied into a bun, ponytail, or braid and had limited movement around the scalp. A loose style was indicated if the hair was tied in bun, ponytail, or braid, but much of the hair around the scalp was free moving. A free hair style was indicted if the participant did not tie up their hair in any fashion. Hair properties were recorded using a categorical approach (Table 1).

**Table 1:** Each participant’s hair was categorized by curl type, style at participation, and tightness of style at the time of participation

Curl type	Style at participation	Tightness of style
Tight curls	Low bun	Free
Curly	Low ponytail	Loose
Wavy	Shorter than 1-inch	Tight
Straight	Down	
	Braid	
	Between 1-inch and shoulder length	

**Fig. 1:**
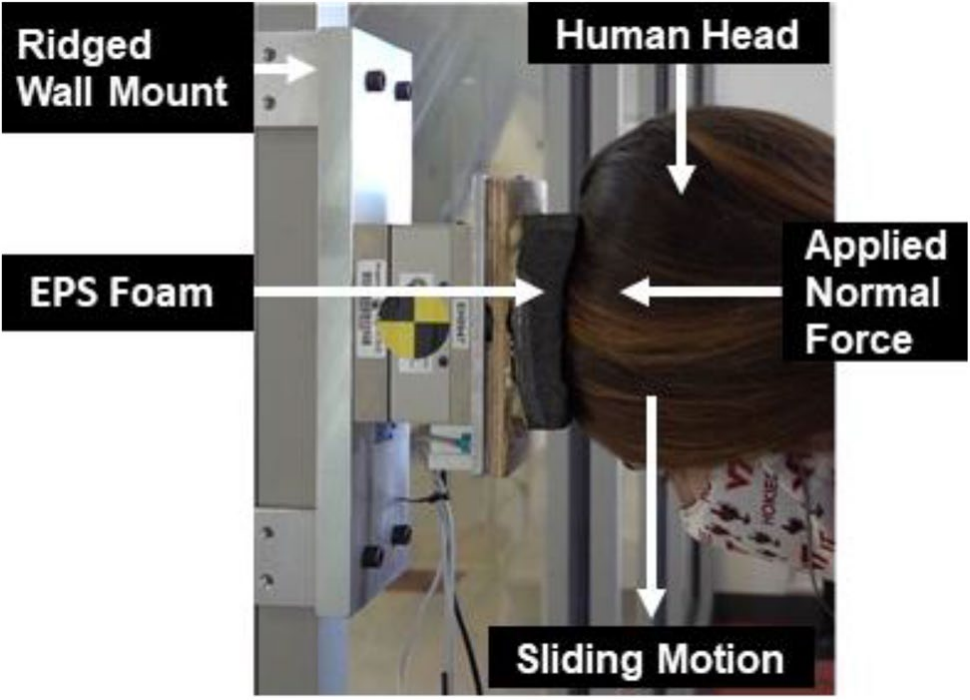
Participants slid their heads nine times along EPS foam while applying three different normal force loads (low, medium, high) to the foam. The EPS foam was rigidly mounted on a trial axial load cell that captured the normal and tangential forces during each trial

From a standing position and a comfortable distance, participants were asked to lean over, place a finger on their hairline, and then line up their finger with the bottom edge of the vertically mounted EPS foam. Participants were then instructed to remove their finger and press their head as much as possible to the EPS foam surface (Fig. 1). Once the participant’s head was fully contacting the EPS foam, they were instructed to apply the appropriate normal force level. Each participant performed a total of nine sliding motions at three participant perceived applied normal force levels (low, medium, and high). Participants were instructed to apply force based on a self-perceived scale varying from 1 to 10, where 1 is barely touching, and 10 is the maximum force they could apply. Participants were instructed that a low applied normal force was two on the scale, the medium force was five to six, and the high normal force was eight to ten on the self-perceived scale. After the participant indicated that they reached the appropriate normal force, the study staff member would instruct the participant to rotate and move their head down to slide their heads along the contour of the padding until their heads are off the padding or the top of their head ends and they cannot slide their heads on the padding anymore. The participant was instructed to make the movement last 3 s, which was verified by a study staff member who would count out loud with a stopwatch. Each trial’s sagittal view was also captured using a video camera.

The applied normal force was the force the subject exerted onto the device along the *z*-axis. The tangential force was defined as the measured *x*- and *y*-axis resultant force [20]. After each participant, the EPS foam was checked for damage and cleaned with a dissentient cloth to remove any residue that may have transferred from the participant hair.

**Fig. 2:**
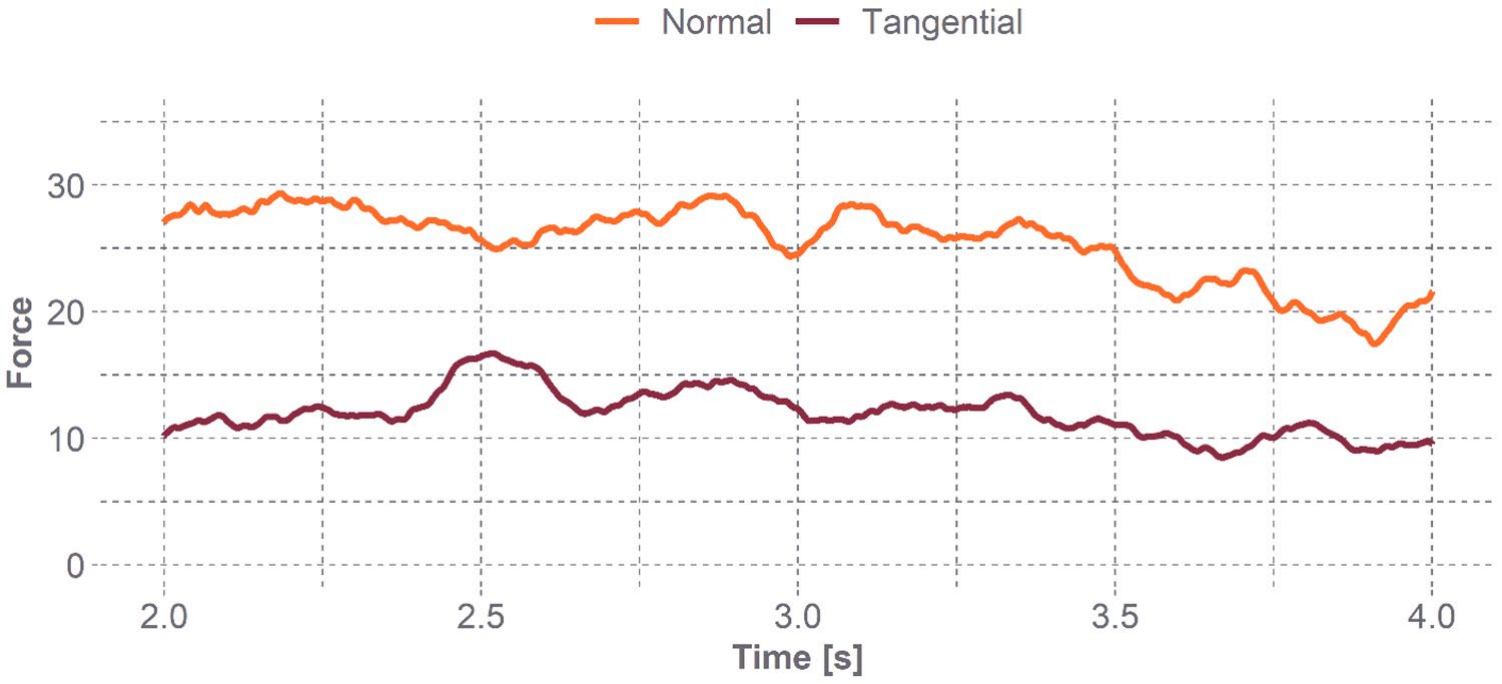
Normal (orange) and tangential (red) force verses time for a single trial. Just after 2.5 s, the participants head started to move along the foam indicating the static COF point. COF was calculated as the tangential force divide by the normal force. Calculated COF over time for a single trial

Normal and tangential forces were collected from the load cell during the nine trials at 20 kHz. The raw force data for each trial were then processed in MATLAB (Mathworks; Natick, Massachusetts, USA). The 3-axis force data were filtered using a 4-pole phaseless lowpass Butterworth filter with a 100 Hz cut-off frequency. Baseline offsets in the signal were corrected for, and the data were smoothed using a 50-ms moving average window (Fig. 2).

**Fig. 3:**
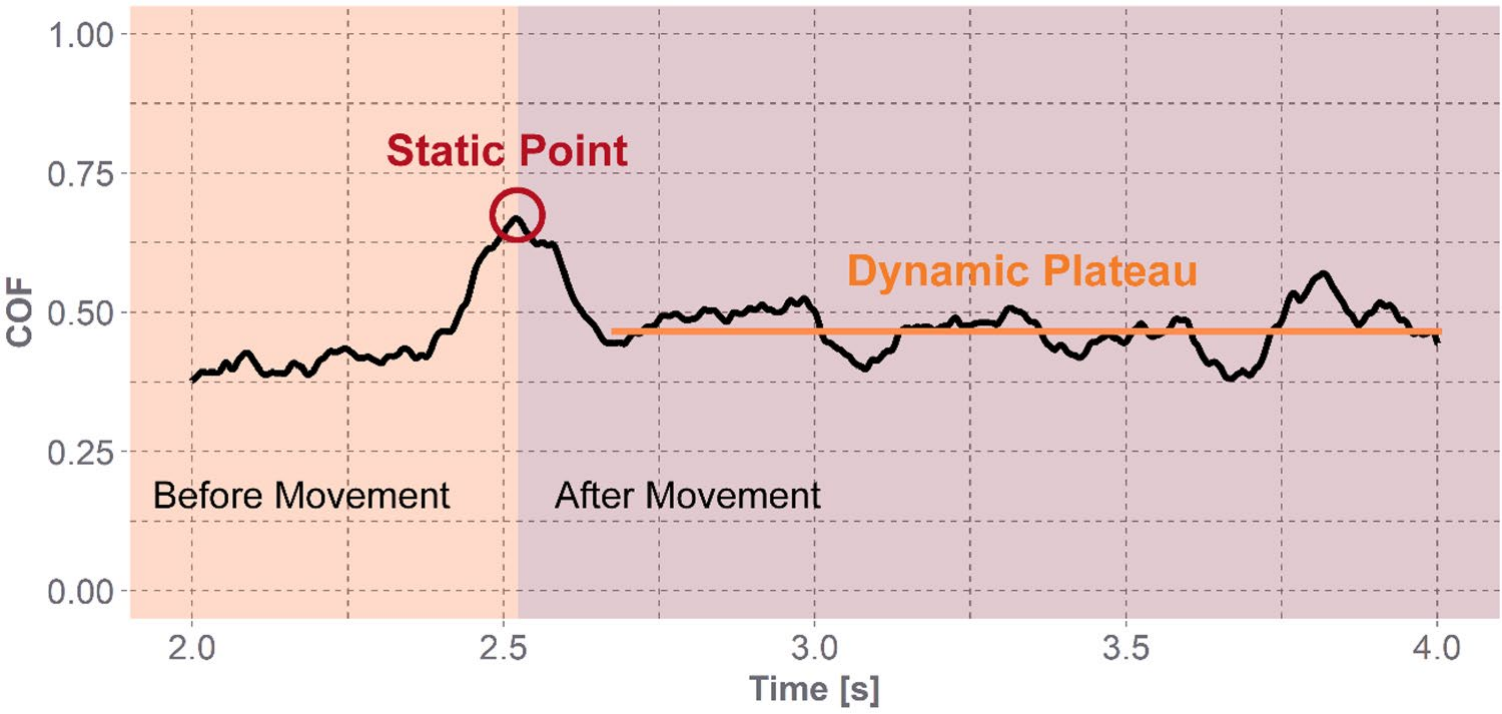
Calculated COF over time for a single trial corresponding data to Fig. 2. The shaded orange region is the start of applied force before movement occurs, and the shaded red region is the start of the sliding motion after overcoming the static COF

For each trial, the COF over time was estimated by dividing the tangential force by the normal force. The static COF was defined as the maximum COF value on the COF vs time trace after force application began just before the movement occurred. Movement was identified by a large decrease in the COF and was confirmed through video analysis. The dynamic COF was defined as the average COF of the plateau region during movement (Fig. 3). The plateau region was also indefinable by a large spike in COF toward the end of the trial that indicated the participant was no longer in contact with the EPS foam, and this was also confirmed with video analysis.

For each applied normal force level, the average static COF, dynamic COF, and applied normal force were computed across the three trials. Each trial was rated based on signal quality. Signals were rated as either low, acceptable, or high quality. A low-quality signal was defined as a signal where static and dynamic friction coefficient features could not be clearly identified from the force signatures or there was a large (5–10 N) drift in the normal force application by the participant. The clarity of the features was also used to define the acceptable, identifiable with video referencing, and high-quality signals, easily identifiable features.

Power-law curves were fit to model static and dynamic COF as a function of applied normal force between all participants, based on the known power-law relationship (Eq. 1) in RStudio (Version 1.2, RStudio; Boston, Massachusetts, USA) [2, 21]. We also fit static and dynamic COF power-law curves separately for males and females. 95% confidence intervals (CI) were computed for each curve using 1000 bootstrap samples.

1 $${\text{COF}} = a*{\text{Applied}}\;{\text{Normal}}\;{\text{Force}}^{b}$$

A biological sex-based effect was added to the exponent of the model to determine the impact of biological sex on model parameters (Eq. 2). Data were fit using a nonlinear least squares regression to define the coefficients and corresponding *p* values and determine the statistical significance of biological sex on the model parameters. A threshold of *p* < 0.05 for the model’s biological sex coefficient was used to determine if biological sex had a significant effect on friction coefficients.

2 $${\text{COF}} = a*{\text{Applied}}\;{\text{Normal}}\;{\text{Force}}^{{b + {\text{sex}}}}$$

Quantitative comparisons of hair properties (curl type, style at time of participation, and tightness of style) at high normal force level were conducted to determine their effect on static COF. Hair properties were compared for the high applied normal force and for only static COF, as real-world impacts occur at kN normal force levels and impact durations are approximately 10 ms [3, 4, 24]. Therefore, comparing the asymptote of the curve at high normal forces for static friction is considered the most appropriate approach.

## Results

The 74 participants ranged in age from 18 to 39 years (Table 2) and were 51% female. Most participants wore their hair in a low ponytail/bun (40%) or had a short hairstyle (45%). Of the 666 samples, 580 were considered acceptable or high quality and used in the analysis. Table 3 depicts the applied normal force levels, means, and standard deviation, for all participants and by biological sex.

**Table 2:** The breakdown of participants by sex and hair properties

Variable	Category	Participants
Biological sex	Males	36
	Females	38
Curl type	Tight curls	7
	Curly	13
	Wavy	20
	Straight	34
Tightness of style	Free	33
	Loose	30
	Tight	11
Style at participation	Shorter than 1″	11
	1″ to shoulder length	23
	Low bun/ponytail	30
	Down	3
	Braid	6
	Other	2

**Table 3:** Applied normal force levels

Applied normal force	All participants	Males	Females
High	80 ± 34	96 ± 32	68 ± 29
Medium	45 ± 21	51 ± 23	39 ± 17
Low	24 ± 12	27 ± 14	21 ± 9

Mean ± standard deviation

**Fig. 4:**
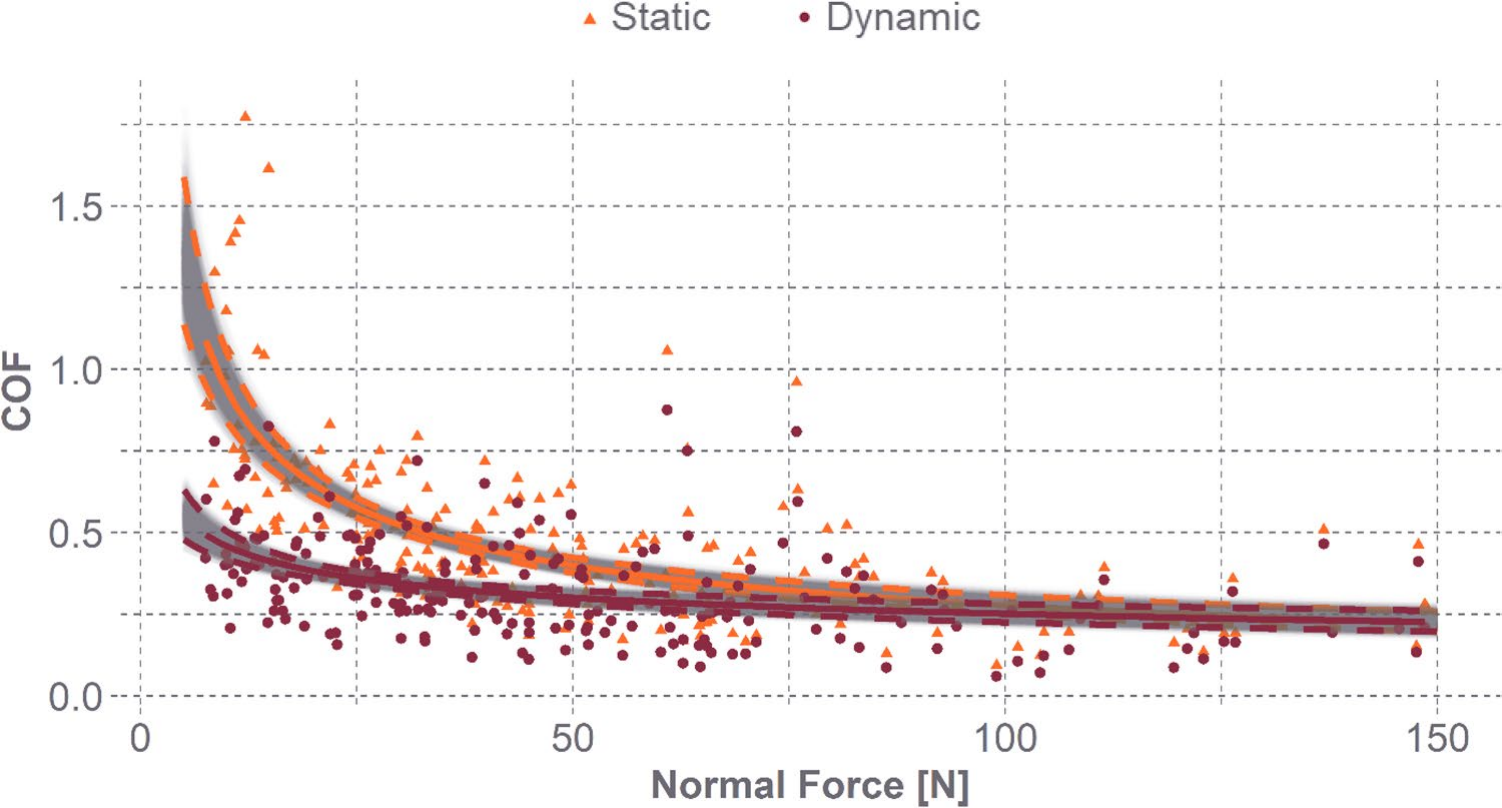
The static (orange) and dynamic (red) COF over applied normal force [N]. Including a mean power-law curve (solid line) and 95% CI curves (dashed lines)

Static and dynamic COF varied with normal force, and higher applied normal forces generated lower COF values (Fig. 4, Table 4). To highlight the decline in COF with normal force, we evaluated each measure at 50 N, the mean normal force applied, and 80 N, the mean high applied normal force. At 50 N, the human head and EPS foam static friction coefficient is 0.39 (95% CI 0.36–0.42), and the dynamic friction coefficient is 0.29 (95% CI 0.27–0.32). At 80 N, the static friction coefficient is 0.32 (95% CI 0.30–0.34) and the dynamic friction coefficient is 0.27 (95% CI 0.26–0.28).

**Table 4:** COF static and dynamic power-law curve parameters

	*a*	*b*
Static COF	2.21 (2.09 to 2.44)	− 0.44 (− 0.44 to − 0.45)
Dynamic COF	0.56 (0.54 to 0.59)	− 0.17 (− 0.16 to − 0.18)

**Fig. 5:**
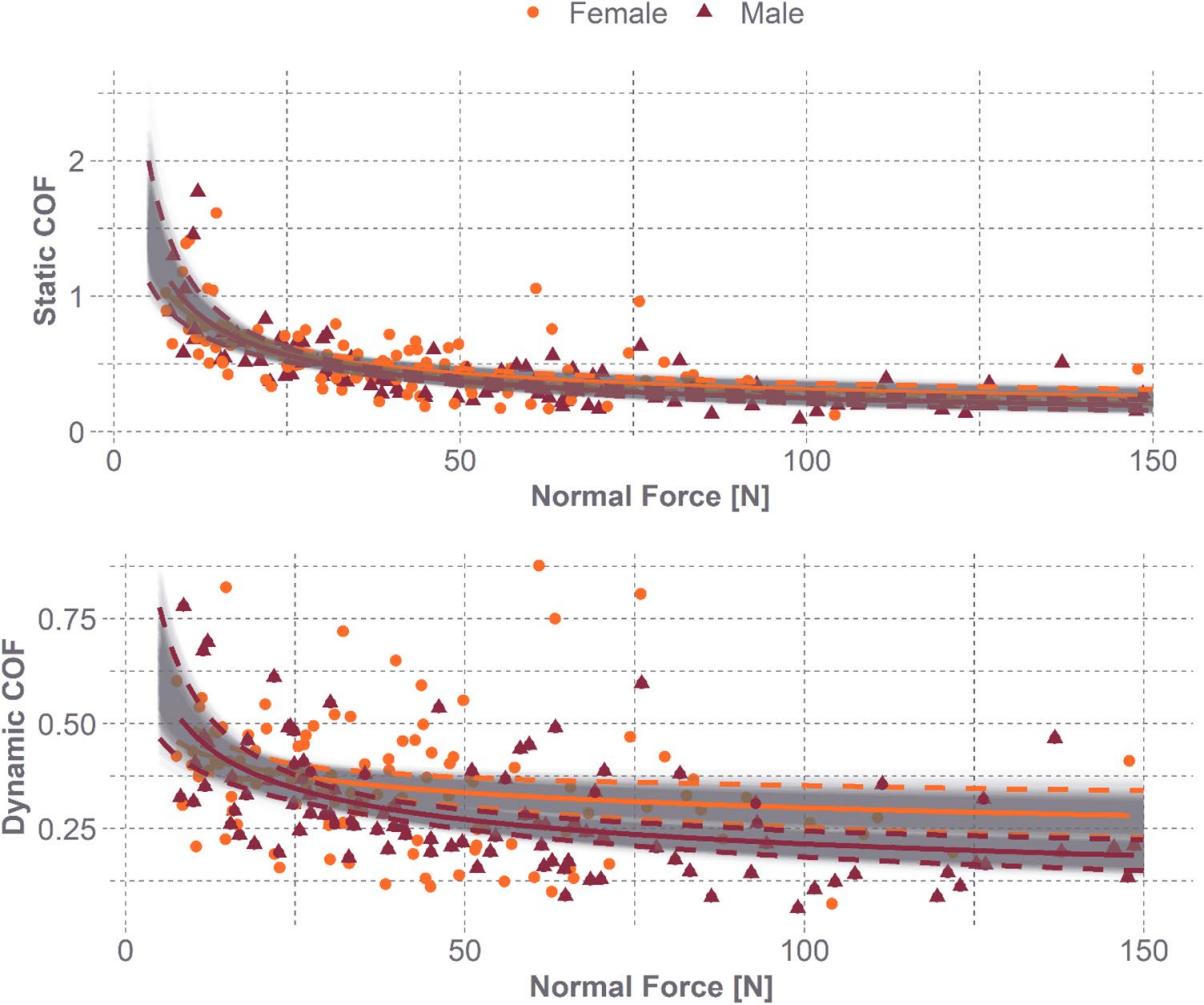
Biological sex comparison for static and dynamic COF vs. applied normal force. Including a mean power-law curve (solid line) and 95% CI curves (dashed lines)

While significant, the effect size was small when accounting for biological sex in the static (*p* = 0.006) and dynamic (*p* = 0.001) friction coefficient models. The static friction coefficient at 80 N for females is 0.34 (95% CI 0.30–0.38) and for males is 0.30 (95% CI 0.27–0.32), a difference of 0.04 with overlapping CI. The dynamic friction coefficient for females is 0.30 (95% CI 0.26–0.35), and for males is 0.24 (95% CI 0.21–0.27), a difference of only 0.06 with overlapping CI (Fig. 5).

**Fig. 6:**
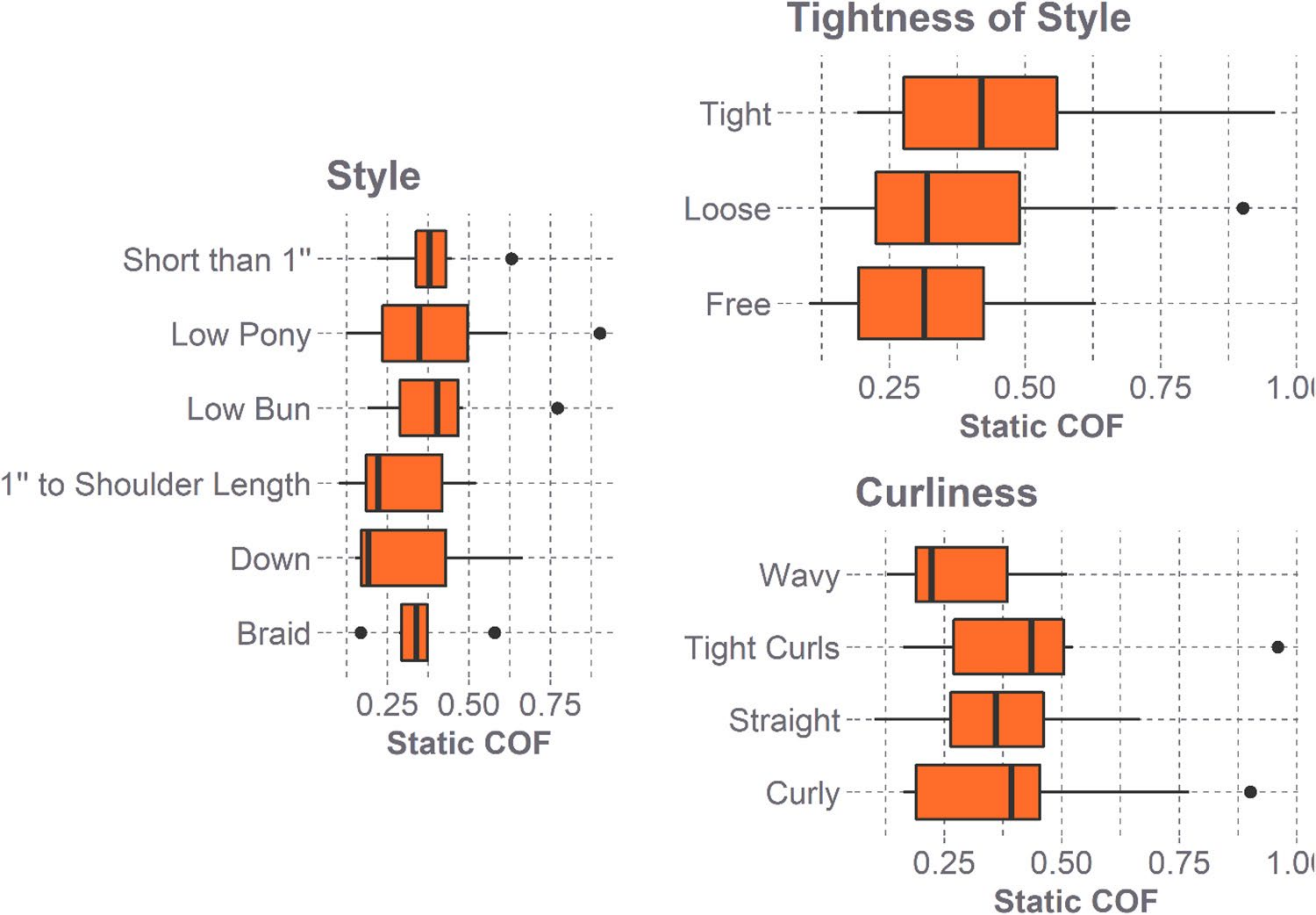
Box plot comparing all participants static COF for the categorical hair properties at a high applied normal force. The hair properties included curl (straight, wavy, curly, tight curls), style at the time of participation (down, braid, low bun, low ponytail, *s*horter than *1*″, longer than *1*″ but shorter than shoulder length*)*, and tightness of style at the time of participation (free, loose, tight)

Participant’s hair properties, including curl type, style at the time of participation, and tightness of style at participation, showed no effect on the static COF at the high applied normal force (Fig. 6).

## Discussion

This study quantified the head and helmet interface static and dynamic COF relative to the normal force. Friction may significantly impact rotational acceleration and the resultant head injury prediction for the head impact test [1, 13]. Friction coefficients between the head and helmet interface have not been thoroughly characterized before, making it hard to determine if headform friction characteristics are biofidelic. Our data also show that biological sex and hair properties do not significantly affect head and helmet interface friction coefficients.

At the average high normal force level, 80 N, the human head had a static COF between 0.30 and 0.34 and a dynamic COF between 0.26 and 0.28. Trotta et al. reported that the static COF ranges between 0.21 and 0.35 averaged over 20–200 N (mean 90 N) falling within the CI of our findings [33]. Trotta et al. also stated that dynamic COF is in the range of static COF [33]. However, we found that static COF is higher than the dynamic COF of 0.26–0.28. Although our study’s static and dynamic friction coefficients are close to the ranges found by Trotta et al., the comparison is hard to make due to the difference in testing methods and sample size. Trotta et al. COF values were reported as an average for the range of normal forces applied (20–200 N) and tested against polyester fabric and not EPS foam [33]. Furthermore, Ebrahimi et al. reported a COF of 0.683 for skin against helmet material which is on the top end of the static COF we found at low applied normal force and over double the static and dynamic COF at 80 N [13]. However, it is essential to note that this was also a different helmet material and further analysis would be needed to determine the difference in interacting materials that aaffect the human head COF. Ebrahimi et al. also did not report the two applied normal forces used, if the friction was static or dynamic, or if it was skin from the head, which made the comparisons to our results vague [13].

Trotta et al. also reported that at higher stroke frequencies (material moving over the head) when applied normal force increased, there was a reduction in COF [33]. This study demonstrates that a power–law relationship can describe the decrease in friction coefficients with increasing normal force when evaluating human head friction coefficients. Seo et al. reported similar trends against other areas of the skin for both dynamic and static friction coefficients [30]. This is likely due to the skin's viscoelastic properties [5, 6, 30], as viscoelastic textiles also display a power–law relationship between normal force and friction coefficients [15, 27]. Based upon this evidence, human head friction coefficients should be evaluated with respect to the normal force using a power–law relationship for dynamic and static friction coefficients.

Although statistically significant, at 80 N, the difference in COF between males and females is small. Only a 0.04 static COF and 0.06 dynamic COF difference was found between biological sexes at 80 N and both had overlapping CI. Furthermore, the variance in the mean static (0.04) and dynamic (0.06) COF between the biological sexes is within the normal variance expected in a human participant study. Given these points, it is probably not necessary to account for biological sex differences; however, the effect of altering COF by this small difference on linear and rotational kinematics during impact is unknown. This should be investigated before deciding how much fine-tuning of COF is required to reasonably recreate real-world impact events. The distribution of static COF overlapped considerably for all hair properties confirming Trotta et al. finding that hair did not affect COF [33]. This study also determined that no specific hair property (curl, style at the time of participation, and tightness of style at the time of participation) affects the static COF.

This study had several limitations. One limitation is that the study population consisted of younger adults (18–39 years old), though age has been shown not to affect skin COF [31]. Another study limitation is that we only evaluated the COF against EPS foam and not against comfort lining materials. Furthermore, in estimating dynamic COF, we did not consider the acceleration of the head during movement. Thus, the dynamic coefficient was an estimate based on normal and tangential force and could overestimate the actual dynamic friction coefficient when accounting for acceleration. However, head impacts in the real world typically last around 10 ms [4, 17] and as a result, static friction would dominate the frictional response. Hence, considering the static friction coefficient is critical when evaluating the biofidelity of the dummy headform. For this study, participants were instructed to slide their heads along the foam for three to four seconds. Therefore, the accelerations had low magnitudes, and we suspect this would only introduce minor errors.

Finally, the main limitation of this study is that the normal applied force levels and strain rates are well below the forces experienced during an impact. Working with human participants, we could not enforce an applied normal force or strain rate close to the level of a head impact event as it may result in injury to the participant. Therefore, the highest normal force level of 80 ± 34 N is substantially below the approximate 5 kN normal force experienced during a bicycle impact [4, 17]. The strain rate used in our study was also comparatively longer, with a duration of 3 s, whereas real-world head impacts typically last around 10 ms [4, 17].

Although the 80 N force is still well below the normal forces experienced by dummy headforms during impact testing, the published friction coefficients for dummy headforms do not state the normal force. Partly due to this, a wide range of COF is reported for dummy headforms. The COF reported for Hybrid III headforms is 1.07 [7] and 0.75 ± 0.06 [33], both well above the human head COF. When a stocking or hair is applied to the Hybrid III headform, Bonin et al. reported a COF of 0.26 or 0.17 [7]. Therefore, the addition of a stocking cap to the Hybrid III headform reduces the COF into the range of the human head. The magnesium EN960 bare headform has reported COF of 0.23 [13], 0.16 ± 0.03 [33], and 0.20 [25]. Based on these reported COF, the bare EN960 headform is slightly below the human head CI of the static or dynamic COF. However, when the EN960 headform is covered in silicon rubber, COF has been reported to increase from 0.78 to 0.81 causing the EN960s headforms friction to be well above the human heads [13, 25]. Currently, there are no reported friction coefficients for the NOCSAE headform. However, each headform COF was found using various testing methods, and there has not been any published COF vs. normal force curves for any headform.

This study compared human head COF across a range of normal forces and reported the power-law curve relationships for static and dynamic COF. Our evaluation of friction coefficients between the head and helmet interface included a larger living sample population and a larger interacting surface area compared to previous studies. Our findings also demonstrate that biological sex and hair properties have little effect on the frictional characteristics of the human head. This study can be used to compare the COF of dummy headforms with that of the human head at defined normal forces. The defined static and dynamic friction coefficients should be used to develop more realistic head impact testing methods, define helmet-head boundary conditions for computer-aided simulations, and aid the optimization and development of helmet designs. Future research should evaluate the COF of commonly used headforms in impact testing to determine their friction biofidelity against the COF found for the human head using similar testing methods. There also should be an evaluation of how headforms with different COF affect oblique impact testing results.

## Abbreviations

CI: Confidence interval
COF: Coefficient of friction
EPS: Expanded polystyrene
MIPS: Multi-directional Impact Protection System
NOCSAE: National Operating Committee on Standards for Athletic Equipment
ODS: Omni-Directional Suspension
SPIN: Shearing Pads INside
